# Efficiency Enhancement in Ocean Thermal Energy Conversion: A Comparative Study of Heat Exchanger Designs for Bi_2_Te_3_-Based Thermoelectric Generators

**DOI:** 10.3390/ma17030714

**Published:** 2024-02-02

**Authors:** Yi-Cheng Chung, Chun-I Wu

**Affiliations:** Department of Mechanical and Mechatronic Engineering, National Taiwan Ocean University, Keelung 20224, Taiwan

**Keywords:** thermoelectric materials, Bi_2_Te_3_, ocean thermal energy conversion, thermoelectric generator, heat exchanger, longitudinal vortex generators, renewable energy, sustainable development, sustainable technology

## Abstract

This research focuses on enhancing the efficiency of Bi_2_Te_3_-based thermoelectric generators (TEGs) in ocean thermal energy conversion (OTEC) systems through innovative heat exchanger designs. Our comparative study uses computer simulations to evaluate three types of heat exchangers: cavity, plate-fins, and longitudinal vortex generators (LVGs). We analyze their impact on thermoelectric conversion performance, considering the thermal energy transfer from warm surface seawater to TEGs. The results demonstrate that heat exchangers with plate-fins and LVGs significantly outperform the cavity heat exchanger regarding thermal energy transfer efficiency. Specifically, plate-fins increase TEG output power by approximately 22.92% and enhance thermoelectric conversion efficiency by 38.20%. Similarly, LVGs lead to a 13.02% increase in output power and a 16.83% improvement in conversion efficiency. These advancements are contingent upon specific conditions such as seawater flow rates, fin heights, LVG tilt angles, and locations. The study underscores the importance of optimizing heat exchanger designs in OTEC systems, balancing enhanced heat transfer against the required pump power. Our findings contribute to a broader understanding of materials science in sustainable energy technologies.

## 1. Introduction

### 1.1. Energy Issues

The continuous progress in technology necessitates an increased demand for energy and the optimization of energy utilization. The predominant source of our energy supply continues to be derived from fossil fuels. Drawing upon current figures from the U.S. Department of Energy and Lawrence Livermore National Laboratory [1,2], it is evident that in the year 2019, a significant proportion of the energy consumed in the United States, specifically 80%, originated from fossil fuel sources such as oil, coal, and natural gas. Throughout the energy conversion process, around 68% of the energy transforms into waste heat, rendering it impractical for efficient utilization. In conjunction with their suboptimal energy conversion efficiency, the combustion of fossil fuels results in the emission of greenhouse gases, exacerbating global warming and giving rise to a host of environmental concerns. Therefore, two important goals for achieving sustainability are to improve the current efficiency of energy conversion and to increase the use of renewable energy sources to cut down on carbon emissions.

### 1.2. Application of Thermoelectric Generators (TEGs) in Waste Heat Recovery

Thermoelectric materials have the distinctive capability of converting thermal energy into electrical energy without an intermediate conversion process. TEGs are composed of multiple thermoelectric couples and are renewable energy technology. In recent years, TEGs have been extensively utilized for waste heat recovery. One potential method of harnessing waste heat produced by car engines involves the installation of TEGs into the exhaust pipe and radiator [3]. The thermal energy generated by the human body temperature can be harnessed and utilized to operate wearable devices using TEGs [4].

Furthermore, it is possible to recover the waste heat produced during the energy-intensive process of cement manufacturing by utilizing TEGs. This approach can effectively improve the energy efficiency of many industrial operations [5]. It is advisable to consult pertinent review papers [1,6,7,8].

### 1.3. Application of Bi_2_Te_3_ TEGs in Ocean Thermal Energy Conversion (OTEC)

TEGs have been demonstrated to have potential applications within the domain of ocean thermal energy [9,10]. Ocean thermal energy is a prominent ocean energy source. OTEC is the technological method employed to exploit the temperature disparity between warm surface seawater and cold deep saltwater to generate power [9]. The Closed-Cycle OTEC (CC-OTEC) system adheres to conventional practices and uses a working fluid with a low boiling point that circulates in a closed loop. This continuous circulation of the fluid enables it to drive a turbine, facilitating energy generation [11].

In 1980, the concept of thermoelectric OTEC was established by M. S. Bohn et al. [12]. The authors also conducted a comparison between thermoelectric OTEC and CC-OTEC. Thermoelectric OTEC presents a range of possible benefits in comparison to CC-OTEC, which include the following: First, the utilization of TEGs in electricity generation has the potential to replace intricate mechanical components such as turbines, thereby simplifying the system and enhancing its reliability and durability. Second, water is utilized as the working fluid, mitigating any leakage and environmental contamination issues. The benefits mentioned above render thermoelectric OTEC a compelling alternative for using ocean thermal energy.

Bismuth telluride (Bi_2_Te_3_) has emerged as a promising thermoelectric material for near-room-temperature applications. Its high Seebeck coefficient and reasonable electrical conductivity allow for the efficient conversion of heat to electrical energy, making Bi_2_Te_3_ ideal for OTEC systems. OTEC exploits the temperature differential between the warmer surface and colder deep seawater to generate power. At approximately 300 K, Bi_2_Te_3_ achieves optimal thermoelectric efficiency and material stability. Although its performance diminishes at higher temperatures, it remains robust at the colder temperatures in OTEC condenser systems. Advancements in reducing lattice thermal conductivity could further enhance Bi_2_Te_3_’s efficiency and cost-effectiveness in OTEC plants, facilitating the production of clean, renewable electricity from minimal ocean temperature gradients. High-quality Bi_2_Te_3_ devices could significantly contribute to sustainable energy solutions.

### 1.4. Design of Heat Exchangers

Although TEGs possess certain advantages, such as their reliability, they are also characterized by limitations in thermoelectric conversion efficiency. The operational basis of TEGs relies on the Seebeck effect, whereby an increase in the amount of heat energy absorbed by TEGs results in a corresponding increase in converting this energy into electrical power [13]. The effective transfer of heat energy to TEGs is contingent upon the design of heat exchangers, which assumes pivotal significance.

The following references are pertinent regarding the scholarly discourse on heat exchangers employing rectangular channels for TEGs. In a study by Kumar et al. (2011), the authors investigated the utilization of TEGs to recover waste heat from internal combustion engines [14]. The research focused on evaluating the thermoelectric conversion efficiency of TEGs across various operating conditions of internal combustion engines. Using numerical simulation data, the authors conducted a comparative analysis of three distinct cross-sectional shapes (hexagonal, triangular, and rectangular) for cavity heat exchangers. Their findings indicate that heat exchangers with rectangular cross-sections exhibit a more homogeneous distribution of flow fields, rendering them well suited for TEG heat exchange applications.

In a study conducted in 2020, Yan et al. employed numerical simulations to examine the effects of various cross-sectional forms of channels (including square, rectangular, triangular, trapezoidal, and octagonal) on the performance of TEGs [13]. The study revealed that rectangular channels exhibited smaller cross-sectional areas at equivalent Reynolds numbers, increasing flow velocities. Consequently, rectangular channels demonstrated the capability to deliver greater output power.

In order to improve the thermal conductivity characteristics of heat exchangers and channels, several investigations have integrated fin configurations into their respective architectures. A study by Weng et al. (2013) investigated using TEGs to extract waste heat from automobile exhaust systems [15]. The researchers developed heat exchangers in the shape of hexagonal prisms, which were explicitly designed to be installed on the exhaust pipe. The input and output ends of the heat exchanger were linked to the exhaust pipe. TEGs were affixed to the external surface of the heat exchanger. At the same time, plate-fins were implemented on the internal side of the heat exchanger in order to augment heat conduction. The study’s findings indicate that the positioning of TEGs impacts their output power. Specifically, TEGs placed closer to the upstream section of the heat exchanger exhibit elevated hot-side temperatures, leading to an increase in output power.

A study conducted by J.-Y. Jang et al. in 2013 examined the utilization of TEGs to recover waste heat from chimneys [16]. In this study, TEGs were affixed to the outside surface of the chimney, while plate-fins were introduced onto the interior surface of the chimney. The study’s findings indicate that the incorporation of plate-fins led to a significant enhancement in the maximum output power of the TEGs, exhibiting a 33-fold increase compared to TEGs without fins. In addition, it was observed that the fins’ quantity and dimensions significantly impacted the power production and conversion efficiency of the TEGs. The augmentation of fin height and quantity resulted in elevated pressure drops. It necessitated greater pump power, causing the progressive enhancements in the net output power and thermoelectric conversion efficiency of the TEGs to plateau.

A study was undertaken by Bai et al. (2014) to investigate the application of TEGs in vehicle waste heat recovery systems [17]. The researchers conducted a comparative analysis of six distinct heat exchanger designs, each with unique internal structures. These structures included an empty cavity, inclined plate-fins, parallel plate-fins, separate plates with holes, a serial plate structure, and a pipe structure. This analysis aimed to assess the influence of these internal structures on the performance of the TEG. The study revealed that, when subjected to comparable engine circumstances, the continuous plate-fin configuration facilitated the movement of exhaust gases along the fins, augmenting the fluid’s trajectory within the heat exchanger. Consequently, increased heat transmission to the TEG was observed, leading to the maximum heat transfer rate. However, it is noteworthy that this design also exhibited the highest pressure drop.

In addition to using conventional fin configurations to enhance the heat transfer efficiency of heat exchangers, the fluid’s inherent fluid dynamic characteristics can influence the heat transfer effectiveness of the heat exchanger. In contrast to laminar flow, turbulence induces vortices that perturb the boundary layer, enhancing heat transfer from the fluid to the wall surface [18]. This approach holds promise for enhancing the heat transfer efficiency of the heat exchanger and further augmenting the thermoelectric conversion capabilities of TEGs. One effective technique for inducing turbulence involves the utilization of vortex generators. These devices can generate transverse and longitudinal vortices within the flow channel, influencing the channel’s heat transfer characteristics [19].

In a study conducted in 2017, Ma et al. employed numerical simulations to examine the thermoelectric–hydraulic characteristics of a heat exchanger utilized in TEGs [20]. The researchers integrated longitudinal vortex generators (LVGs) into the heat exchanger and subsequently observed the formation of intricate three-dimensional vortices in the downstream region of the LVGs. The vortices generated by LVGs resulted in an augmented pressure drop within the heat exchanger. However, the energy expended by the pump to counteract this pressure drop was found to be less than the power output of the TEG. Hence, implementing LVGs positively impacted the TEG’s overall net output power.

Based on the findings of the current study, it has been observed that while few research works have examined and compared the influence of heat exchangers equipped with plate-fins or pin fins on the operational efficiency of TEGs [21,22], there is currently a dearth of studies that specifically investigate and contrast the impact of plate-fins and LVGs on the thermoelectric conversion performance of TEGs. Consequently, drawing upon the earlier literature analysis, the present study examines the influence of rectangular flow channel heat exchangers equipped with plate-fins and LVGs on the thermoelectric conversion efficiency of TEGs in OTEC systems.

This paper presents a novel approach in the field of sustainable energy. Its uniqueness lies in its focus on improving the efficiency of Bi_2_Te_3_-based thermoelectric generators (TEGs) in ocean thermal energy conversion (OTEC) systems through innovative heat exchanger designs. This paper employs computer simulations to evaluate three types of heat exchangers, revealing that specific designs notably increase thermoelectric conversion efficiency. This study contributes significantly to the broader understanding of materials science in sustainable energy technologies, highlighting the critical role of optimizing heat exchanger designs in OTEC systems. By balancing enhanced heat transfer against the required pump power, the findings offer valuable insights for developing more effective and sustainable energy conversion methods.

## 2. Numerical Methods

### 2.1. Structure, Materials, and Dimensional Design

**Figure 1:**
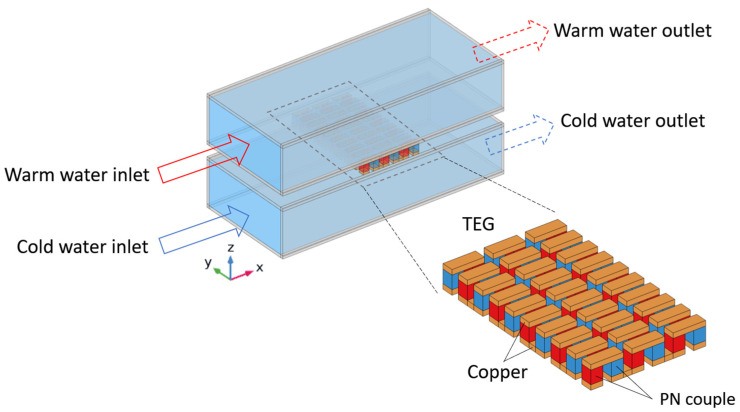
Schematic diagram of the TEG installed in a cavity heat exchanger.

This study examines three types of heat exchangers: cavity heat exchangers, heat exchangers with plate-fins, and heat exchangers equipped with LVGs. Figure 1 illustrates the structural diagram of the TEG integrated into heat exchangers with vacant cavities. The heat exchanger is subjected to the flow of surface warm seawater at a temperature of 25 °C on its upper side. The heat exchanger’s lower side experiences the flow of deep cold saltwater at a temperature of 4 °C. The TEG is positioned between the two heat exchangers in the intermediary space. The TEG is composed of 30 sets of thermocouples, wherein each set comprises a p-type semiconductor (bismuth telluride, Bi_2_Te_3_), an n-type semiconductor (bismuth telluride, Bi_2_Te_3_), and a connecting conductor (copper). The p-type and n-type semiconductors possess cross-sectional dimensions measuring 1.4 mm × 1.4 mm, while their heights are 1.6 mm. The copper thickness measures 0.6 mm, while the distance separating the two legs of the thermocouple is 1 mm. The flow channel is constructed using alumina as the material. It possesses specific dimensions, including an internal width of 23 mm, an internal height of 10 mm, a length of 53.4 mm, and a thickness of 0.7 mm. The material properties of the TEG and flow channel, such as the Seebeck coefficient, electrical conductivity, thermal conductivity, density, and heat capacity, have been obtained from the literature [23,24]. The material properties of seawater, such as dynamic viscosity, thermal conductivity, density, and heat capacity, are obtained from [25].

**Figure 2:**
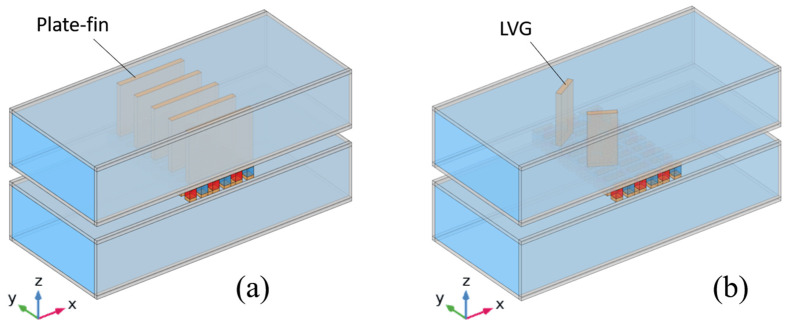
Schematic diagram of the TEG installed in (**a**) a heat exchanger with plate-fins and (**b**) a heat exchanger with LVGs.

The figures presented in Figure 2a,b show the structural schematics of the TEG when installed on heat exchangers equipped with plate-fins and LVGs, respectively. This study examines the positioning of plate-fins in the midstream area of the top side of the heat exchanger, right above the TEG. Copper is utilized as the fin material to optimize the heat transfer process to the TEG. The plate-fins in the x-direction have been designed to have the same length as the TEG, guaranteeing that they adequately cover the area directly above the TEG. The plate-fins possess a thickness of 1 mm. In future investigations, the height of the plate-fins will be modified to 1, 0.75, 0.5, and 0.25 times the height of the heat exchanger. This adjustment examines the influence of varying fin heights on the performance of the TEG.

**Figure 3:**
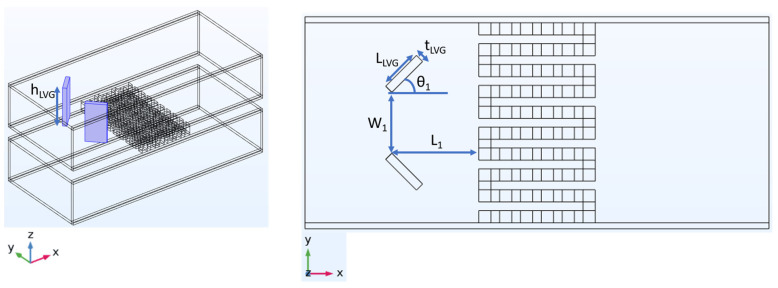
Position, angle, and other dimensional parameters of the LVG.

The dimensional characteristics of the heat exchanger equipped with LVGs are shown in Figure 3. The LVGs are made of copper, with a length of 5 mm and a thickness of 1 mm. The vertical dimension of the LVGs is equivalent to the vertical dimension of the heat exchanger. The spacing between the LVGs is 7 mm. In this research, the position of the LVGs will be adjusted to the upstream (L_1_ = 10 mm) and midstream (L_1_ = 0 mm) of the heat exchanger. Furthermore, the experimental study will involve altering the inclination angle of the LVGs (θ_1_ = 30, 45, 60, 120, 135, 150 degrees) in order to examine the influence of the LVGs’ positions and angles on the vortex effect and, subsequently, on the performance of the TEG.

### 2.2. Governing Equations

The hypothetical conditions for the numerical simulation research are as follows: The fluid entering the heat exchanger exhibits steady-state behavior, meaning that its variables remain constant over time. Additionally, the fluid is incompressible, meaning its density remains constant under the given conditions. Furthermore, the flow of the fluid can be characterized as fully developed, indicating that its velocity profile has reached a stable state. A turbulence model is employed. For the thermodynamic parameters of the system, specifically the heat exchanger and TEG, we focused exclusively on their inherent properties, disregarding the impacts the influences of radiation and convection. Furthermore, in evaluating the thermoelectric properties of the TEG materials, we concentrated on their intrinsic characteristics, omitting considerations of electrical and thermal contact resistances at their surfaces. The governing equations for the system are structured into two primary categories: those applicable to the fluid dynamics and those relevant to the solid constituents. For the fluid, under steady-state conditions, the continuity equation, momentum equation, and energy equation are as follows [13,20]:

Continuity equation:

(1) $$\rho \nabla \cdot \vec{u}=0$$

Navier–Stokes equation:

(2) $$\rho \left(\vec{u}\cdot \nabla \right)\vec{u}=-\nabla p+\mu \nabla^{2}\vec{u}$$

Energy conservation equation:

(3) $$\rho c_{p}\vec{u}\cdot \nabla T+\nabla \cdot \vec{q}=Q$$

where ρ, μ, and $c_{p}$ are the fluid density, dynamic viscosity, and specific heat, respectively; $\vec{u}$ is the velocity vector; p is the pressure; T is the temperature; $\vec{q}$ is the heat flux caused by conduction and radiation, and under conditions without considering radiation effects, $\vec{q}=k_{f}\nabla T$, where $k_{f}$ is the thermal conductivity of the fluid; Q is the heat generated by internal heat sources, whereby if there are no internal heat sources, then Q equals zero. Through the continuity equation and momentum equation, the fluid velocity and pressure can be solved, and then by substituting the velocity into the energy equation, the fluid temperature can be solved.

The solid can be divided into general materials and thermoelectric materials. For general materials like tube walls, fins, and copper conductors, heat is transferred by conduction, and the steady-state heat conduction equation is

(4) $$\nabla \cdot \left(k_{s}\nabla T\right)=0$$

where $k_{s}$ is the thermal conductivity of the general material (tube walls, fins, connecting conductors).

For thermoelectric materials, under steady-state conditions, the energy conservation equation can be written as follows [7,26,27]:

(5) $$\nabla \cdot {\vec{q}}_{\mathrm{TE}}=Q_{\mathrm{Joule}}$$

Moreover, the steady-state charge conservation equation is

(6) $$\nabla \cdot \vec{J}=0$$

where ${\vec{q}}_{\mathrm{TE}}$ is the heat flux at the surface of the thermoelectric material; Joule heating $Q_{\mathrm{Joule}}$ can be written as $Q_{\mathrm{Joule}}={\left|\vec{J}\right|}^{2}/\sigma$; $\vec{J}$ is the current density.

The coupled governing equations that dictate thermoelectric behavior [7,26,27] are

(7) $${\vec{q}}_{\mathrm{TE}}=ST\vec{J}-k\nabla T$$

(8) $$\vec{J}=\sigma \left(\vec{E}-S\nabla T\right)$$

where S, k, and σ are the Seebeck coefficient, thermal conductivity, and electrical conductivity of the thermoelectric material, respectively; $\vec{E}$ is the electric field, which can also be expressed by the electric potential gradient −∇V so that the second governing equation can be rewritten as $\vec{J}=-\sigma \left(\nabla V+S\nabla T\right)$.

Substituting the coupled thermoelectric governing equations into the energy and charge conservation equations yields provides us with the following:

(9) $$\nabla \cdot \left(ST\vec{J}\right)-\nabla \cdot \left(k\nabla T\right)=\frac{{\left|\vec{J}\right|}^{2}}{\sigma }$$

(10) ∇·(σ∇V)+∇·(σS∇T)=0

The above two equations are the coupling relationships for temperature and electric potential. When a specific current is input, the electric potential and temperature distributions in the thermoelectric material can be solved from the equations; when a specific temperature is input, the current and electric potential distributions can be solved.

### 2.3. Boundary Conditions

The boundary conditions for the numerical simulation in this study are as follows:

- The inlet of the heat exchanger is set as a fully developed flow with specified average velocity and temperature.
- The outlet of the heat exchanger has a pressure of zero.
- The outer surfaces of the heat exchanger are adiabatic.
- Except for the hot and cold end interfaces, the TEG surfaces are adiabatic.
- All solid walls have no-slip boundaries.
- In the TEG module, the terminal of the first thermocouple on the far right is grounded, and other boundaries of the TEG are electrically insulated.

### 2.4. Performance Evaluation Parameters

In order to evaluate the influence of structural design on TEG performance, the TEG evaluation parameters [8,28,29] are defined as follows. The internal resistance $R_{\mathrm{pn}}$ of a thermocouple can be written as

(11) $$R_{\mathrm{pn}}=\frac{\rho_{p}L_{p}}{A_{p}}+\frac{\rho_{n}L_{n}}{A_{n}}$$

The thermal conductance Κ of the thermocouple can be written as

(12) $$K=\frac{\lambda_{p}A_{p}}{L_{p}}+\frac{\lambda_{n}A_{n}}{L_{n}}$$

where $\rho_{p}$ and $\rho_{n}$ are the electrical resistivities of the p-type and n-type thermoelectric materials; $L_{p}$, $L_{n}$ are the lengths of the p-type and n-type thermoelectric materials; $A_{p}$, $A_{n}$ are the cross-sectional areas of the p-type and n-type thermoelectric materials; $\lambda_{p}$, $\lambda_{n}$ are the thermal conductivities of the p-type and n-type thermoelectric materials.

The Seebeck coefficient of the thermocouple can be written as

(13) $$\alpha_{\mathrm{pn}}=\alpha_{p}-\alpha_{n}$$

where $\alpha_{p}$, $\alpha_{n}$ are the Seebeck coefficients of the p-type and n-type thermoelectric materials.

For the TEG, the internal resistance $R_{\mathrm{TEG}}$ and output voltage $V_{\mathrm{oc}}$ of the TEG can be written as

(14) $$R_{\mathrm{TEG}}=N\left(R_{\mathrm{pn}}+2\frac{\rho_{c}L_{c}}{A_{c}}\right)$$

(15) $$V_{\mathrm{oc}}=N\alpha_{\mathrm{pn}}\left(T_{h}-T_{c}\right)$$

where N is the number of thermocouples in the TEG; $\rho_{c}$, $L_{c}$, $A_{c}$ are the electrical resistivity, length, and cross-sectional area of the connecting conductors; $T_{h}$ and $T_{c}$ are the temperatures of the TEG’s hot and cold sides. To estimate the output power P of the TEG, an external load resistance $R_{L}$ is connected to form a closed-loop current

(16) $$I=\frac{V_{\mathrm{oc}}}{R_{\mathrm{TEG}}+R_{L}}$$

(17) $$P=I^{2}R_{L}$$

From the above two equations, the output power can be written as

(18) $$P={\left(\frac{V_{\mathrm{oc}}}{R_{\mathrm{TEG}}+R_{L}}\right)}^{2}R_{L}$$

When the external load resistance equals the internal resistance, the maximum output power $P_{\max}$ occurs, which can be written as

(19) $$P_{\max}=\frac{{V_{\mathrm{oc}}}^{2}}{4R_{\mathrm{TEG}}}$$

The net output power $W_{\mathrm{net}}$ is

(20) $$W_{\mathrm{net}}=P_{\max}-W_{\mathrm{pump}}$$

where $W_{\mathrm{pump}}$ is the pumping power required for the heat exchanger, which can be written as $W_{\mathrm{pump}}=v_{\mathrm{in}}A_{\mathrm{tube}}\Delta p$. $v_{\mathrm{in}}$ is the inlet velocity, $A_{\mathrm{tube}}$ is the flow channel cross-sectional area, and Δp is the pressure drop between outlet and inlet. The heat absorption $Q_{h}$ at the thermoelectric unicouple hot side can be written as

(21) $$Q_{h}=\alpha_{\mathrm{pn}}IT_{h}+Κ\left(T_{h}-T_{c}\right)-\frac{1}{2}I^{2}R_{\mathrm{TEU}}$$

The heat absorption is composed of three parts on the right-hand side of the above equation, which are Peltier heat (first term), thermal conduction heat (second term), and Joule heat (third term), where $\alpha_{\mathrm{pn}}$ is the Seebeck coefficient of the thermoelectric unicouple, *I* is the current, Κ is the thermal conductance of the thermoelectric unicouple, $T_{h}$ and $T_{c}$ are the temperatures of the thermoelectric unicouple’s hot and cold sides, and $R_{\mathrm{TEU}}$ is the internal resistance of the thermoelectric unicouple. The total heat absorption $Q_{\mathrm{total}}$ can be written as $Q_{\mathrm{total}}=NQ_{h}$. By taking the ratio of the net output power to the total heat absorption, the thermal conversion efficiency *η* of the TEG can be estimated as

(22) $$\eta =\frac{W_{\mathrm{net}}}{Q_{\mathrm{total}}}$$

### 2.5. Convergence Test

This study uses the finite element method (FEM) software COMSOL 5.5 [30] for numerical simulations. Since the number of meshes affects the calculation results of FEM, convergence tests are performed first on the three structures to be analyzed before conducting the research. Table 1 shows the results of the convergence test, comparing the relative errors of calculation results with different mesh densities. In order to balance solution accuracy and required computational resources, the appropriate number of meshes used in this study is as follows: about 390,000 for TEG installed in the cavity heat exchanger, about 1.05 million for TEG installed in the plate-fin heat exchanger, and about 560,000 for TEG installed in the heat exchanger with LVG. The relative error of TEG installed in the cavity heat exchanger is less than 3.6%, the relative error of TEG installed in the plate-fin heat exchanger is less than 0.8%, and the relative error of TEG installed in the heat exchanger with LVG is less than 0.4%.

## 3. Results and Discussion

The efficiency of the TEG during the analytical procedures undertaken in this investigation are outlined as follows: Initially, the average input velocity should be adjusted to ensure consistency. Subsequently, the thermal flow characteristics and voltage distribution of the three structures should be examined. Following this, the average input velocity of the warm seawater should be modified to investigate the impact of the three structures on the thermoelectric conversion performance of the TEG under varying Reynolds number operating conditions.

### 3.1. Thermal Flow Analysis

**Figure 4:**
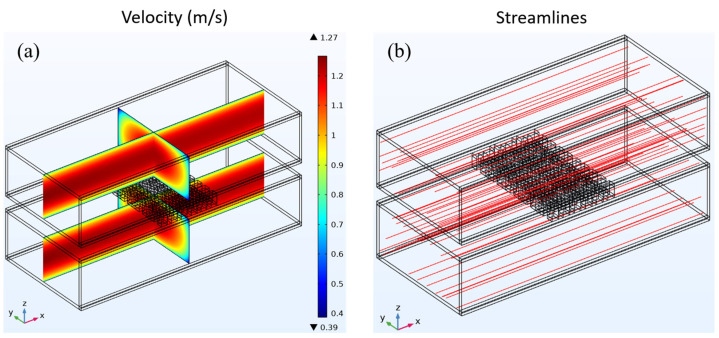
(**a**) Fluid velocity distribution and (**b**) streamline distribution in a cavity heat exchanger.

Initially, it is established that the average input velocity at the heat exchanger intake remains constant, with both the warm and cold seawater exhibiting an average velocity of 1 m/s. Subsequently, an examination of the fluid dynamics parameters of the three heat exchangers is conducted. The fluid velocity distribution and streamline distribution of the cavity heat exchanger are depicted in Figure 4. The velocity distribution depicted in Figure 4a demonstrates that a uniform velocity distribution may be observed along the flow channel direction owing to the fully developed input flow.

As a consequence of viscous processes, the fluid close to the tube wall adheres to the wall, forming a velocity boundary layer. Consequently, the velocity in the vicinity of the wall surface is diminished. In the cavity heat exchanger, the flow lines are parallel to the direction of the flow channel. Figure 4b shows the spread of the streamline.

**Figure 5:**
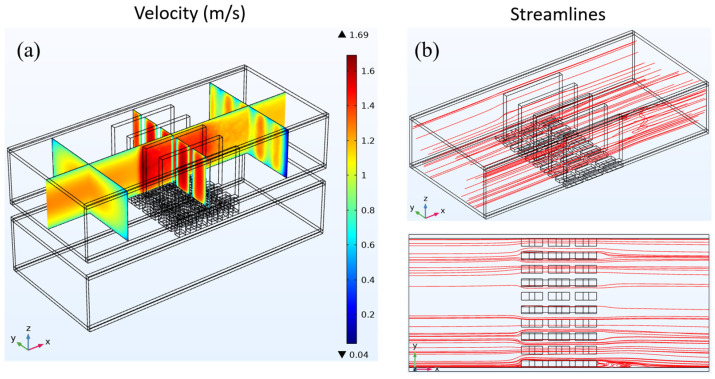
(**a**) Fluid velocity distribution and (**b**) streamline distribution in a heat exchanger with plate-fins.

Figure 5 depicts fluid velocity distribution and streamline patterns throughout the plate-fin heat exchanger. In order to enhance clarity in illustrating the impact of fins on the fluid, the velocity and streamlined distributions exclusively depict the outcomes of the upper heat exchanger. As depicted in Figure 5a, the velocity distribution demonstrates that the fluid entering the heat exchanger exhibits a fully developed flow characterized by a discernible velocity boundary layer near the tube wall. When the fluid comes into contact with the fins in the heat exchanger, the flow velocity between the fins is enhanced due to the reduced cross-sectional area through which the fluid can pass. Moreover, due to the alteration in velocity, the initial velocity boundary layer experiences disruption, resulting in a reduction in thickness for both the upper and lower tube wall velocity boundary layers. As depicted in the streamline distribution illustrated in Figure 5b, when encountering the fins within the heat exchanger, the fluid maintains its forward motion over the surfaces of the fins. This facilitates the potential heat conduction from the fluid to the underlying TEG through the fins.

**Figure 6:**
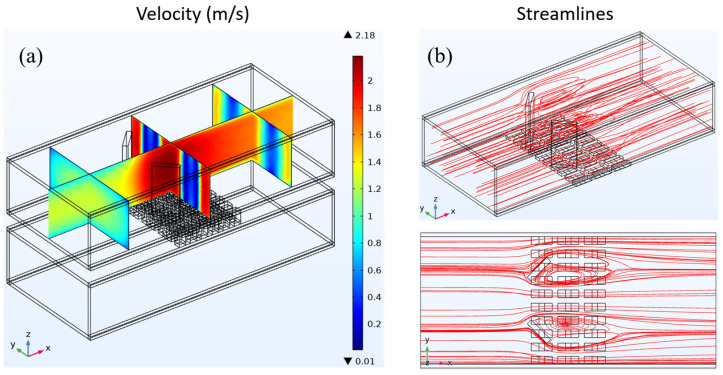
(**a**) Fluid velocity distribution and (**b**) streamline distribution in a heat exchanger with LVG.

Figure 6 shows how the fluid’s speed and streamlines are spread out in the heat exchanger that has LVG. As depicted in Figure 6a, the velocity distribution exhibits a pattern akin to the study conducted on the preceding two constructions. Notably, in the upstream region of the heat exchanger, reduced velocities close to the tube wall are detected, which signifies the presence of a velocity boundary layer. As the fluid traverses the LVG within the heat exchanger, it experiences discernible alterations in its velocity. The regions with greater velocities exhibit concentrations in the central area of the LVG as well as on both sides of the tube wall.

Conversely, the regions with lower velocities are mainly concentrated behind the LVG. The velocity fluctuations indicate the alterations occurring within the velocity boundary layer. As the fluid traverses the LVG, the velocity boundary layer experiences a notable reduction in thickness. The distribution of the streamline is depicted in Figure 6b. When the fluid passes through the LVG, certain flow lines exhibit the formation of three-dimensional vortices in the wake of the LVG. The analysis of the streamline distribution and velocity distribution reveals that the presence of vortices leads to a notable decrease in velocity within the corresponding region.

In contrast, areas devoid of vortex production exhibit higher velocities. The vortices generated by the LVG have a dual effect on the boundary layer, impacting both the velocity boundary layer and the temperature boundary layer. This results in an enhancement of the heat transfer effect, as documented in reference [19]. Creating vortices facilitates heat transfer to the TEG below, which impacts the TEG’s thermoelectric conversion efficiency.

**Figure 7:**
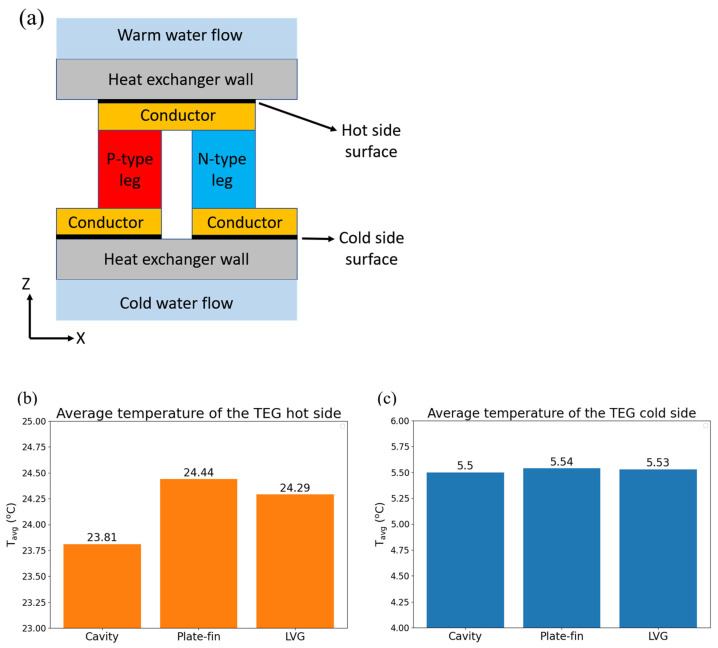
(**a**) The x-z cross-sectional view of a single TEG couple and average temperature of (**b**) the hot end and (**c**) the cold end of TEG and the contact surface with the tube wall in three types of heat exchangers.

**Figure 8:**
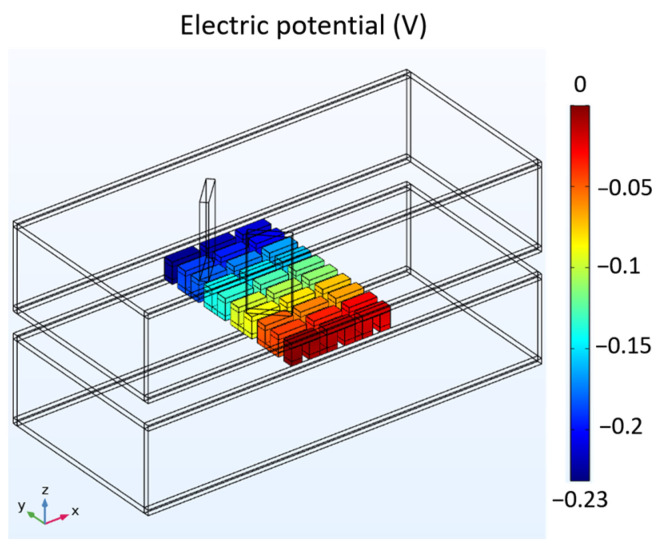
Voltage distribution of TEG installed in the heat exchanger with LVG.

Figure 7 depicts the mean temperature of the hot side and cold side of the TEG and the temperature of the contact surface with the wall in three different configurations: the cavity heat exchanger, the plate-fin heat exchanger, and the heat exchanger with LVG. Based on the comparative analysis of the three heat exchangers, it is observed that the heat exchangers equipped with plate-fins and LVG exhibit higher temperatures of the hot side of the TEG than the cavity heat exchanger. This observation suggests that these two heat exchangers can significantly augment the fluid’s heat transfer capacity to the hot side of the TEG. The fin plates have the most pronounced heat transfer efficacy among the various components. The voltage distribution of the TEG installed in the heat exchanger with LVG is shown in Figure 8. The temperature gradient between its hot and cool sides can generate the voltage differential in the TEG. The voltage distribution chart reveals that the structural configurations of the three heat exchangers can induce a voltage disparity within the TEG. The preceding data represent the outcomes of a comparative analysis conducted under the condition of an average seawater velocity of 1 m/s. In the subsequent section, the present study will examine various flow velocities. Subsequently, the voltage differential will be employed for subsequent computations to assess the thermoelectric conversion efficiency of the TEG.

### 3.2. TEG Performance Analysis

#### 3.2.1. The Impact of Reynolds Number (at the Hot End) on TEG Performance

**Figure 9:**
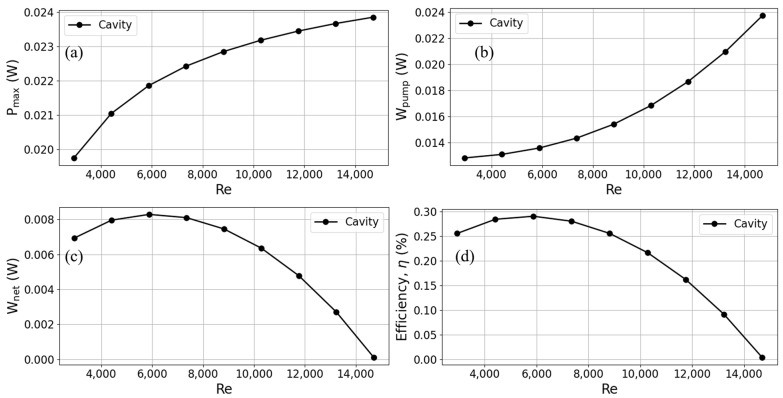
Trends of (**a**) maximum output power, (**b**) pump power, (**c**) net output power, and (**d**) thermoelectric conversion efficiency with Reynolds number for TEG installed in a cavity heat exchanger.

To comprehend the impact of the Reynolds number on the operation of TEGs, this research manipulates the mean input velocity of warm surface seawater at the intake to alter the fluid’s Reynolds number. When comparing turbulent and laminar flows in channel flows, it is seen that the velocity adjustment varies between 0.2 m/s and 1 m/s, which corresponds to Reynolds numbers ranging from 2940.2 to 14701. In this study, we examine the impact of variations in Reynolds number on the performance of a cavity heat exchanger in a TEG. The characteristics assessed about thermoelectric conversion encompass maximum output power, pumping power, net power, and thermal conversion efficiency. The findings are depicted in Figure 9. As depicted in Figure 9a, a rise in the Reynolds number results in a higher fluid input velocity, leading to an augmented flow of warm seawater via the hot side of the TEG per unit time.

Consequently, this increased flow rate enhances the heat transfer to the TEG, resulting in an amplified output power of the TEG. The growth rate in output power exhibits a progressive leveling off when Reynolds numbers reach higher values. Conversely, when the input velocity is heightened, there is a noticeable rise in the pressure differential between the inlet and outlet. This, in turn, leads to a significant escalation in the pumping power required at high Reynolds numbers, as depicted in Figure 9b. As the Reynolds number grows, there is a progressive increase in the output power. However, the growth rate in pumping power gradually surpasses that of output power.

Consequently, the net output power of the TEG reaches a peak and then experiences a slow decline, as illustrated in Figure 9c. Hence, at a Reynolds number of 5880.4, when the velocity exhibits a slow increase to approximately 0.4 m/s, it is observed that the TEG attains its maximum thermal conversion efficiency. As the Reynolds number progressively rises, there is a notable escalation in pumping power, resulting in a gradual decline in the thermal conversion efficiency of the TEG, as depicted in Figure 9d.

#### 3.2.2. Heat Exchanger with Plate-Fins: The Impact of Fin Height on TEG Performance

**Figure 10:**
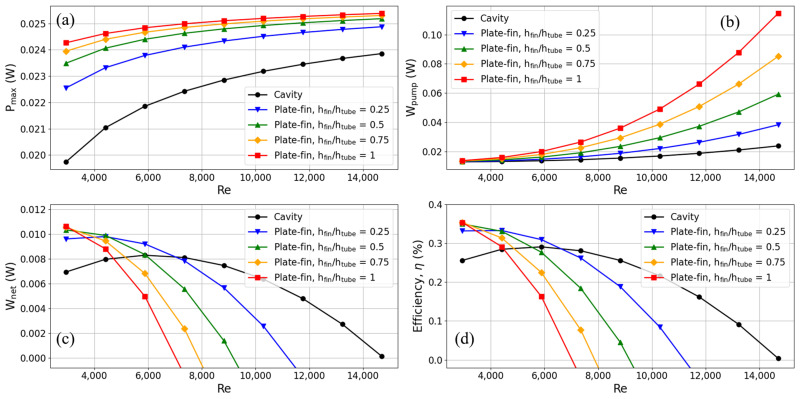
Trends of (**a**) maximum output power, (**b**) pump power, (**c**) net output power, and (**d**) thermoelectric conversion efficiency with Reynolds number for TEG installed in a heat exchanger with plate-fins.

The following section looks at the thermoelectric conversion parameters of TEGs on heat exchangers with plate-fins, as seen in Figure 10. Distinct lines show heat exchangers with varying plate-fin heights and cavity heat exchangers. For heat exchangers with plate-fins, the results show that the output power of TEGs is much higher than in cavity heat exchangers.

Based on the findings of the analysis, it can be observed that fins of varying heights show efficacy in augmenting the output power, as depicted in Figure 10a. The fins with a height equal to 0.25 times the height of the flow channel had the least significant enhancement impact. In comparison to the cavity heat exchanger, the heat exchanger equipped with fins that are 0.25 times the height of the flow channel exhibited a 14.23% enhancement in output power when operating at a flow velocity of 0.2 m/s (corresponding to a Reynolds number of 2940.2). Similarly, at a flow rate of 1 m/s (Reynolds number 14,701), the observed increase in output power was 4.28%. Moreover, it should be noted that an increase in fin height leads to a corresponding increase in output power. This can be attributed to the thermal conduction phenomenon shown by the fins, which facilitates the efficient transmission of thermal energy from the fluid to the TEG. The heat exchanger, including fins of the same height as the flow channel, exhibited the most significant improvement at a flow rate of 0.2 m/s. This improvement resulted in a 22.92% increase in output power. The utilization of fins resulted in an augmentation of the output power; nonetheless, it concurrently elevated the flow resistance within the channel, hence inducing a notable escalation in pump power, as depicted in Figure 10b. As the Reynolds number escalated, the rate of augmentation in output power exhibited a growing inability to match the pace of escalation in pump power. The net output power of the TEG crossed over at a certain point when the heat exchanger with plate-fins was compared to the cavity heat exchanger, as shown in Figure 10c. As illustrated in Figure 10d, the TEG’s thermoelectric conversion efficiency pattern mirrors that of its net output power. When the flow speed was less than 0.5 m/s, equal to a Reynolds number of 7350.5, using plate-fins made the thermoelectric conversion work better. 

On the other hand, when flow rates were higher than 0.5 m/s, the cavity heat exchanger had a higher thermoelectric conversion efficiency. This is because the fins used in it used more pump power. When different fin heights were tested on thermoelectric conversion efficiency at a flow rate of 0.2 m/s, it was found that fins with a height equal to the flow channel height had the highest efficiency (an increase of 38.20% compared to the cavity heat exchanger). Nonetheless, when the flow rate was elevated to 0.3 m/s, there was a notable rise in the pump power required for fins that matched the height of the flow channel. Consequently, this led to a considerable reduction in thermoelectric conversion efficiency. Currently, fins with a height of 0.25 times the flow channel’s height have superior thermoelectric conversion efficiency (an increase of 16.92% compared to the cavity heat exchanger).

#### 3.2.3. Heat Exchanger with LVGs: The Impact of LVG Angle and Position on TEG Performance

Based on earlier research, adding plate-fins to heat exchangers has made TEGs more efficient at converting heat into electricity than cavity heat exchangers. The subsequent phase involves analyzing the influence of implementing LVGs within the heat exchanger regarding the thermoelectric conversion efficiency of TEGs. The study aims to investigate the impact of the position of the LVGs on the creation of vortices. Specifically, the scenarios of LVG placement in the upstream (L_1_ = 10 mm) and midstream (L_1_ = 0 mm) of the heat exchanger will be examined with vortex generation. Furthermore, the vortex’s inclination angle also affects the production of the LVGs by the vortex. Therefore, this study aims to examine the generation of vortices at various inclination angles (θ_1_ = 30, 45, 60, 120, 135, and 150 degrees).

**Figure 11:**
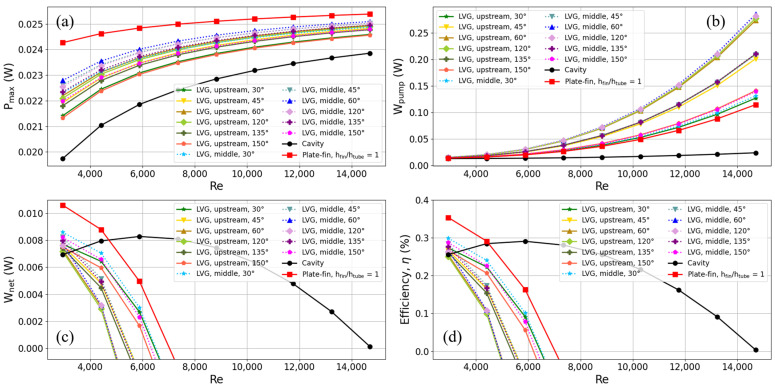
Trends of (**a**) maximum output power, (**b**) pump power, (**c**) net output power, and (**d**) thermoelectric conversion efficiency with Reynolds number for TEG installed in a heat exchanger with LVG. The cavity heat exchanger, the heat exchanger with plate-fins (where the fin height is equal to the height of the flow channel), and the heat exchanger with varied LVG inclination angles are represented by solid lines of different colors.

The main goal of this study is to find out what happens to the efficiency of thermoelectric conversion when LVGs are put upstream of the heat exchanger. The outcomes of this investigation are presented in Figure 11. The results demonstrate that when the flow rate increases from 0.2 m/s (corresponding to a Reynolds number of 2940.2) to 1 m/s (corresponding to a Reynolds number of 14,701), the heat exchanger equipped with LVGs exhibits a greater output power compared to the cavity heat exchanger. This observation suggests that the vortices generated by LVGs significantly enhance heat transfer to the TEG.

In contrast to the heat exchanger employing plate-fins, the heat exchanger, including LVGs, exhibits a comparatively lower output power. This observation implies that the plate-fins utilized in the heat exchanger investigated in this work demonstrate superior heat transfer efficiency, while the LVGs present potential for enhancement. When examining the output power at various inclination angles of the LVG, it is evident that the impact is less pronounced at both small and large angles (30 and 150 degrees). Conversely, at angles of 60 and 120 degrees, a greater output power is detected, with the most significant being recorded at 60 degrees. When the LVG inclination angle is set to 60 degrees, the output power of the heat exchanger increases by 12.41% at a flow rate of 0.2 m/s and by 4.55% at a flow rate of 1 m/s, as compared to the cavity heat exchanger.

Figure 11b presents the pump power findings. The power consumption of the pump in the heat exchanger employing LVGs exceeds that of both the cavity heat exchanger and the heat exchanger equipped with plate-fins. The power required for pumping at short and large angles (30 and 150 degrees, respectively) is significantly smaller. The flow resistance that the LVGs induce increases as the inclination angle of the LVG approaches 90 degrees (such as 60 or 120 degrees), necessitating a significant increase in the pump power required for the heat exchanger. The subsequent analysis centers on the influence of LVG on the net output power and thermoelectric conversion efficiency of TEGs, as depicted in Figure 11c,d, respectively.

In contrast to the heat exchanger equipped with plate-fins, the heat exchanger incorporating LVGs exhibits a reduced output power for the TEG and an increased power need for the pump. Consequently, the net output power of the heat exchanger with LVGs is lower. The thermoelectric conversion efficiency has a similar pattern to that of the net output power. When LVGs are utilized at specified angles, the heat exchanger with LVGs at a flow velocity of 0.2 m/s exhibits a better thermoelectric conversion efficiency than the cavity heat exchanger. When the inclination angle of the LVG is set to 120 and 60 degrees, it increases the demand for pump power, thus leading to a decrease in the thermoelectric conversion efficiency compared to that of the cavity heat exchanger. However, the efficiency is improved at inclination angles of 30, 45, 135, and 150 degrees. The thermoelectric conversion efficiencies are in descending order: 30, 45, 150, and 135 degrees. The thermoelectric conversion efficiency of the TEG is 6.87% higher than that of the cavity heat exchanger when the flow rate is set to 0.2 m/s, and the LVG angle is set to 30 degrees. However, the results show that using LVGs at both small and large angles improves thermoelectric conversion efficiency, with smaller angles (30 to 45 degrees) working better. As the angle approaches 90 degrees, the thermoelectric conversion efficiency utilizing LVGs deteriorates.

The LVGs were then moved to the middle of the heat exchanger (L_1_ = 0 mm), and this study compares this configuration to the thermoelectric conversion characteristics, where the LVGs were placed in the upper part of the system (L_1_ = 10 mm). The dashed lines in Figure 11 depict the outcomes obtained from placing LVGs in the midstream of the heat exchanger. When comparing the outcomes of LVGs located in the upstream and midstream sections of the heat exchanger at identical inclination angles, it is evident that power production is superior when LVGs are positioned in the midstream region. The result shows that positioning LVGs in the midstream section of the heat exchanger results in 4.2%, 3.2%, 2.7%, 2.1%, 2.5%, and 3.1% increases in maximum power output, compared to upstream placement, under identical inclination angles of 30, 45, 60, 120, 135, and 150 degrees, respectively. This phenomenon is because the vortices produced by the LVGs in the midstream region are positioned precisely above the TEG, facilitating heat transmission to the TEG. Furthermore, the observed disparity in pump power between LVGs located in the midstream and upstream regions is not statistically significant.

Consequently, LVGs positioned in the midstream exhibit a comparatively greater thermoelectric conversion efficiency. When the flow rate is set at 0.2 m/s and the LVG inclination angle is adjusted to 30 degrees, precisely positioned in the midstream, the thermoelectric conversion efficiency of the TEG exhibits a notable improvement of 16.83% compared to the cavity heat exchanger. Furthermore, LVGs positioned upstream demonstrate a discernible increase of 6.87% compared to the cavity heat exchanger.

Table 2 summarizes the main calculation results in this study, including the maximum efficiency, power, and heat flux for three types of heat exchangers at a flow velocity of 0.2 m/s. Compared to the cavity heat exchanger, the results show that plate-fins increase TEG output power by approximately 22.92% and enhance thermoelectric conversion efficiency by 38.20%. Similarly, LVGs positioned in the midstream lead to a 13.02% increase in output power and a 16.83% improvement in conversion efficiency.

## 4. Conclusions

This study employs numerical simulations to design and compare three types of heat exchangers: cavity, plate-fins, and LVGs. The TEG is positioned in a configuration sandwiched between two heat exchangers. Its primary function is to harness electrical energy by exploiting the temperature difference between the warmer surface seawater and the colder deep seawater. The potential application of this technology in OTEC systems as a substitute for conventional OTEC systems comprising intricate components is worth considering. This study examines the fluid velocity, streamline distribution, temperature, and other thermal flow properties of three heat exchangers.

Additionally, it investigates the thermoelectric conversion performance of the TEG installed in these heat exchangers at various Reynolds numbers. The analyzed parameters include maximum, pump, and net output power and thermoelectric conversion efficiency. The findings are succinctly described as follows:

1. It has been seen that when the fluid speed and Reynolds number rise in a cavity heat exchanger, the TEG’s output power also rises. However, it is essential to note that the needed pump power also grows in tandem. Consequently, this relationship gives rise to a downward parabolic trend in thermoelectric conversion efficiency. The optimal efficiency of the TEG is attained close to a flow velocity of 0.4 m/s, corresponding to a Reynolds number of 5880.4. As mentioned above, the findings suggest that a compromise between output power and pumping power must be made to attain optimal thermoelectric conversion efficiency, underscoring the need to select an adequate flow velocity.
2. Researchers looked at flow speed, streamlines, and temperature inside heat exchangers and found that heat exchangers with plate-fins and LVGs are better at moving heat from warm seawater to the hot end of the TEG than cavity heat exchangers.
3. Using a heat exchanger with plate-fins increases the TEG’s output power by making it easier for heat to move through the fins. However, it is essential to note that this configuration also leads to an increase in pump power. Thus, as the Reynolds numbers rise, the TEG’s net power and thermoelectric conversion efficiency slowly drop compared to what can be achieved with a cavity heat exchanger. At Reynolds numbers below 0.5 m/s (specifically, Reynolds number 7350.5), it has been seen that the cavity heat exchanger is less efficient at turning heat into electricity than the heat exchanger with plate-fins. When compared to the cavity heat exchanger, where the heat exchanger has fins that are the same height as the flow channel and a flow velocity of 0.2 m/s (corresponding to a Reynolds number of 2940.2), both output power (22.92% increase) and thermoelectric conversion efficiency (38.20% increase) are much better. Additionally, it is demonstrable that there is a direct relationship between the Reynolds number and the pump power necessary to overcome the flow resistance that the fins impose. For example, lowering the height of the fins to 0.25 times the height of the flow channel can increase thermoelectric conversion efficiency.
4. When a heat exchanger with LVGs is used, the TEG’s output power increases because the LVGs create torque waves. However, this approach necessitates a higher pump power, resulting in a notable reduction in net and thermoelectric conversion efficiency as the Reynolds number increases. At Reynolds numbers below a certain threshold, such as 0.2 m/s (corresponding to a Reynolds number of 2940.2), the heat exchanger equipped with LVGs may exhibit a thermoelectric conversion efficiency that surpasses that of the cavity heat exchanger.
5. The inclination angle of the LVGs affects the thermoelectric conversion performance of the TEG. The thermoelectric conversion efficiency at various angles, ranked in descending order, is as follows: 30, 45, 150, 135, 60, and 120 degrees. The findings of this study suggest that LVGs operating at both small and large angles have superior thermoelectric conversion efficiency. Specifically, LVGs operating at slight angles, such as 30 and 45 degrees, demonstrate higher performance. As the angle approaches 90 degrees, there is an observed increase in the power demand of the pump, resulting in a decrease in the efficiency of thermoelectric conversion. When comparing the cavity heat exchanger to the heat exchanger with LVGs (with LVGs positioned upstream at an angle of 30 degrees), it was seen that at a flow rate of 0.2 m/s (corresponding to a Reynolds number of 2940.2), the latter exhibited a higher TEG output power, showing an increase of 8.46%. Additionally, the heat exchanger with LVGs showed an increase in thermoelectric conversion efficiency of 6.87%.
6. The placement of LVGs at various locations inside the heat exchanger, including upstream (L_1_ = 10 mm) and midstream (L_1_ = 0 mm), affects the thermoelectric conversion performance of the TEG. The difference in pump power needed for LVGs upstream and midstream is not very big, but the midstream configuration has more output power, which makes thermoelectric conversion more efficient. When the flow rate is 0.2 m/s, equal to a Reynolds number of 2940.2, the heat exchanger with LVGs (with the LVGs placed midstream at an angle of 30 degrees) works better than the cavity heat exchanger. Specifically, the heat exchanger with LVGs exhibits a notable increase in TEG output power of 13.02% and a corresponding improvement in thermoelectric conversion efficiency of 16.83%.

## Figures and Tables

**Table 1:** Convergence test for three structures.

Channel Type	Number of Elements	Relative Errors			
		*P*_max_ (W)	*Q*_h_ (W)	∆*p* (Pa)	*W*_net_ (W)
Cavity	161,902	−0.16%	−0.06%	8.23%	−7.94%
	388,308	0.03%	0.01%	3.58%	−3.25%
	575,438	—	—	—	—
Plate-fin	417,119	−0.36%	−0.15%	−12.60%	−16.63%
	1,047,071	−0.20%	−0.09%	0.49%	0.72%
	1,571,362	—	—	—	—
LVG	239,673	−0.29%	−0.12%	−0.54%	−0.58%
	555,767	−0.32%	−0.14%	−0.21%	−0.20%
	1,634,565	—	—	—	—

**Table 2:** Summary of maximum efficiency, power, and heat flux for three types of heat exchangers.

Channel Type (v_in,warm_ = 0.2 m/s)		*P*_max_ (W)	*Q*_total_ (W)	*η* (%)
Cavity		1.97 × 10^−2^	2.71	0.26
Plate-fin (h_fin_/h_tube_ = 1)		2.43 × 10^−2^	3.00	0.35
LVG (θ_1_ = 30°)	Upstream	2.14 × 10^−2^	2.82	0.27
	Midstream	2.23 × 10^−2^	2.88	0.30

## Data Availability

The original contributions presented in this study are included in the article; further inquiries can be directed to the corresponding author.
